# A stochastic network-based model to simulate farm-level transmission of African swine fever virus in Vietnam

**DOI:** 10.1371/journal.pone.0247770

**Published:** 2021-03-03

**Authors:** Hu Suk Lee, Krishna K. Thakur, Long Pham-Thanh, Tung Duy Dao, Anh Ngoc Bui, Vuong Nghia Bui, Huy Nguyen Quang

**Affiliations:** 1 International Livestock Research Institute (ILRI), Hanoi, Vietnam; 2 Department of Health Management, Atlantic Veterinary College, University of Prince Edward Island, Charlottetown, PE, Canada; 3 Epidemiology Division, Department of Animal Health, Hanoi, Vietnam; 4 National Institute of Veterinary Research, Hanoi, Vietnam; Plum Island Animal Disease Center, UNITED STATES

## Abstract

African swine fever virus is highly contagious, and mortality rates reach up to 100% depending on the host, virus dose, and the transmission routes. The main objective of this study was to develop a network-based simulation model for the farm-level transmission of ASF virus to evaluate the impact of changes in farm connectivity on ASF spread in Vietnam. A hypothetical population of 1,000 pig farms was created and used for the network-based simulation, where each farm represented a node, and the connection between farms represented an edge. The three scenarios modelled in this way (baseline, low, and high) evaluated the impact of connectivity on disease transmission. The median number of infected farms was higher as the connectivity increased (low: 659, baseline: 968 and high: 993). In addition, we evaluated the impact of the culling strategy on the number of infected farms. A total of four scenarios were simulated depending on the timing of culling after a farm was infected. We found that the timing of culling at 16, 12, 8, and 6 weeks had resulted in a reduction of the number of median infected farms by 81.92%, 91.63%, 100%, and 100%, respectively. Finally, our evaluation of the implication of stability of ties between farms indicated that if the farms were to have the same trading partners for at least six months could significantly reduce the median number of infected farms to two (95^th^ percentile: 413) than in the basic model. Our study showed that pig movements among farms had a significant influence on the transmission dynamics of ASF virus. In addition, we found that the either timing of culling, reduction in the number of trading partners each farm had, or decreased mean contact rate during the outbreaks were essential to prevent or stop further outbreaks.

## Introduction

African swine fever (ASF) is an infectious disease in pigs caused by a DNA virus of the *Asfarviridae* family, genus *Asfivirus* [1]. The virus is transmitted through direct contact with infected pigs or indirect contact via, consumption of contaminated feed, or through fomites [2, 3]. It is a contagious disease with mortality rates reaching up to 100% depending on the host, virus dose, and the transmission routes [4]. The disease can have substantial economic impacts on countries, affecting international pig and pork trade and reducing pig production in affected areas. It is, therefore, a threat to global food security [5]. The disease is endemic in most of the sub-Saharan countries and emerged in the Caucasus, Eastern Europe, and Baltic countries in 2007 [6]. In August 2018, the first outbreak in Asia was reported in the northeast of China [7] and subsequently spilled over to other Asian countries, including Vietnam [8, 9].

In Vietnam, the first occurrence of ASF was reported in February 2019 in backyard pig farms in Hung Yen province, about 250 km from the border with China and 50 km from Hanoi [10]. Since then, ASF outbreaks have been reported in all 63 provinces, and approximately 20% (6 million) of the pig population has been culled [9]. As of March 2020, no outbreaks were reported in 35 provinces for more than 30 days. Poor biosecurity in smallholder farms (accounting for 70% of pig production) was the main contributing factor to the rapid spread across the country over a short period, resulting in huge economic losses for the pig industry [11].

Simulation models are a critical tool for policymakers to help them better understand the impact of disease outbreaks and to inform decision making on cost-effective control strategies (e.g., vaccination and movement controls) [12–14]. Another commonly used method in veterinary epidemiology to evaluate livestock movement patterns and their role on infectious disease transmission is social network analysis (SNA) [15–17]. A number of studies have been conducted to demonstrate the role of contact patterns among farms using SNA [18–21]. These studies provided insight into the impact of network characteristics on disease spread in populations.

In Vietnam, recent studies on pig movement structures provided better insights into which farm production systems were likely to play an important role in the spread of infectious diseases in pigs [11, 22, 23]. However, to our knowledge, no studies have been conducted to simulate the spread of ASF among farms using their contact network structure in Vietnam. The main objective of this study, therefore, was to develop a network-based simulation model for the farm-level transmission of ASF virus to evaluate the impact of changes in farm connectivity on ASF spread in Vietnam.

## Materials and methods

### Study population

A hypothetical population of 1,000 pig farms was created and used for the network-based simulation, where each farm represented a node, and the connection between farms represented an edge. These farms were classified into three production types: 1) small <100 pigs, 2) medium ≥100, and < 1000 pigs 3) large farms ≥1000 pigs [24]. In Vietnam, the proportion of small, medium, and large production type farms is approximately 70–75%, 20–25%, and <5%, respectively. For the sake of simplicity, each production type was assigned 70% (small farms), 25% (medium farms), and 5% (large farms), respectively, which is summarized in Table 1 [25].

**Table 1:** Study population, parameters, values and assumptions used for simulation of network model of ASF transmission.

Parameters	Value	Reference
Total farms (n)	1,000	
Small	700 (70.0%)	(Lapar et al., 2003)
Medium	200 (25.0%)	
Large	50 (5.0%)	
Overall mean degree	0.75	Assumption
Infectious duration	Maximum 52 weeks	Assumption
Transmission probability		
Direct contact*	0.6	(Guinat et al., 2016)
Indirect contact		
Small farms	0.6	contaminated products (e.g. swill) are the main source of infection for which the indirect contact transmission probability for small and medium farm was assumed to be same as that for direct contact
Medium farms	0.6	
Large farms	0.006	

* the max value is used

### Model parameters

Three key parameters are essential for the network model: 1) overall mean degree (estimated number of edges between farms at each time step) and mean degree by farm type; 2) transmission probability (TP) (risk of transmission given contact with an infected farm); and, 3) mean contact rate (frequency of contacts per farm per unit time). These parameters were estimated from previous studies or were based on expert opinion and assumptions. It is well understood that ASF virus in small farms in Vietnam is mainly transmitted through indirect contact (e.g., swill feeding and human/vehicle movements). Therefore, TPs for direct contact for all three farm types were considered the same value (0.6) based on previous studies while indirect contact TP for the small and medium farms were also assigned the same value as direct contact TP due to their low biosecurity status. The indirect contact TP for large farms was estimated based on previous studies and assumptions (expert opinions). The mean contact rates per farm type combination were used from the study used to simulate the between farm spread of porcine reproductive and respisratory syndrome virus in Vietnam [23]. In addition, we had to create the new mean contact rate per week between farms as the network model we used was not able to distinguish between direct and indirect contacts (Table 2). Since the magnitude of direct/indirect mean contact rates was different due to the associated TPs, the new mean combined contact rate per week was generated using the following formula:

Meancombinedcontactrateperweek=Directmeancontactrate+(IndirectmeancontactratexTPforindirectcontactbyfarmtype)

**Table 2:** Description of contact rates of pig farms used for ASF transmission simulation model.

Contact groups (Source-Destination)	Mean contact rate/week (Lee et al., 2019)		
	Direct	Indirect	New combined contact rate*
Small farms → Small farms	0.072	0.282	**0.241**
Medium farms → Medium farms	0.073	0.271	**0.236**
Large farms → Large farms	-	3.5	**0.021**

*formula: direct contact rate + indirect contact rate × transmission probability

### Network model structure

In order to evaluate the impact of network structures on between-farm level transmission of ASF virus, a stochastic computer network simulation model was developed using the package “EpiModel” [26] in R-language (R Development Core Team 2020). EpiModel first uses separable-temporal exponential-family random graph models (STERGMs) to estimate and simulate complete dynamic networks based on network-level patterns of density, degree, dissolution rate, and other network features influencing edge formation and dissolution. It then simulates the spread of an infection over the simulated network based on user-defined disease transmission parameters and contact rates. At first, A susceptible-infected (SI) disease spread model was developed for ASF virus using the network model structure (S1 Fig). It was assumed that all farms were susceptible at the beginning of the model, and none of the pigs had resistance to ASF virus. Once the first pig in a farm became infected, the entire farm was considered infectious. In the model, we hypothesized that at the beginning, one medium farm was randomly selected to be infectious and the same farm-initiated infection in the following iterations. The rest of the susceptible farms were allowed to become infectious, as the simulation progressed, and remain so either until the end of the study period or when they were culled. The model was run over 200 iterations for 52 weeks, which was long enough to cover the pig production life cycle (5–8 months) in Vietnam. In addition, for simplicity, it was assumed that a continuous flow (CF) system was used in all farms in our network models, this system is followed by most of the small and some medium farms in Vietnam, where they continually introduce replacement animals from different farms with unknown disease status and also implement minimal biosecurity practices. All-in-all-out system (AIAO), is followed by some medium and most large farms in Vietnam.

### Scenarios and sensitivity analysis

A total of 7 scenarios were developed as follows: 1) the network connectivity based on the mean degree (baseline, low and high); 2) intervention scenarios (R1-R4) for SI-Removed (R) models to evaluate the impact of stamping out the infected farms on the epidemic spread. For the connectivity scenarios, our baseline scenarios began with 375 total edges (mean degree = 0.75) and the assumption that 75% of farms were involved in animal movement. After that, low (250 edges, mean degree = 0.5) and high (500 edges, mean degree = 1) connectivity network scenarios were generated to evaluate the impact of connectivity between farms on the model outcomes. We developed the SIR models to assess the impacts of stamping out of infected farms on the epidemic spread. A total of four scenarios were created that incorporated culling at 6, 8, 12 and 16 weeks since the first farm became infected during the study period.

For sensitivity analysis, we evaluated the impact of the weekly mean contact rate by a reduction of 25%, 50% and 75%, respectively, to the baseline model. In addition, we also evaluated the impact of stability of ties between farms with first the farms changing their trading partners frequently (networks with higher dissolution rate) for the baseline model-this is a very common practice for most of the small and medium farms in Vietnam and then having comparatively stable trading partnerships between the farms. Finally, the number of median infected farms from each scenario was compared to the baseline model. From all simulated network models, we calculated the number of median infected farms with 5th and 95th percentiles.

## Results

**Fig 1:**
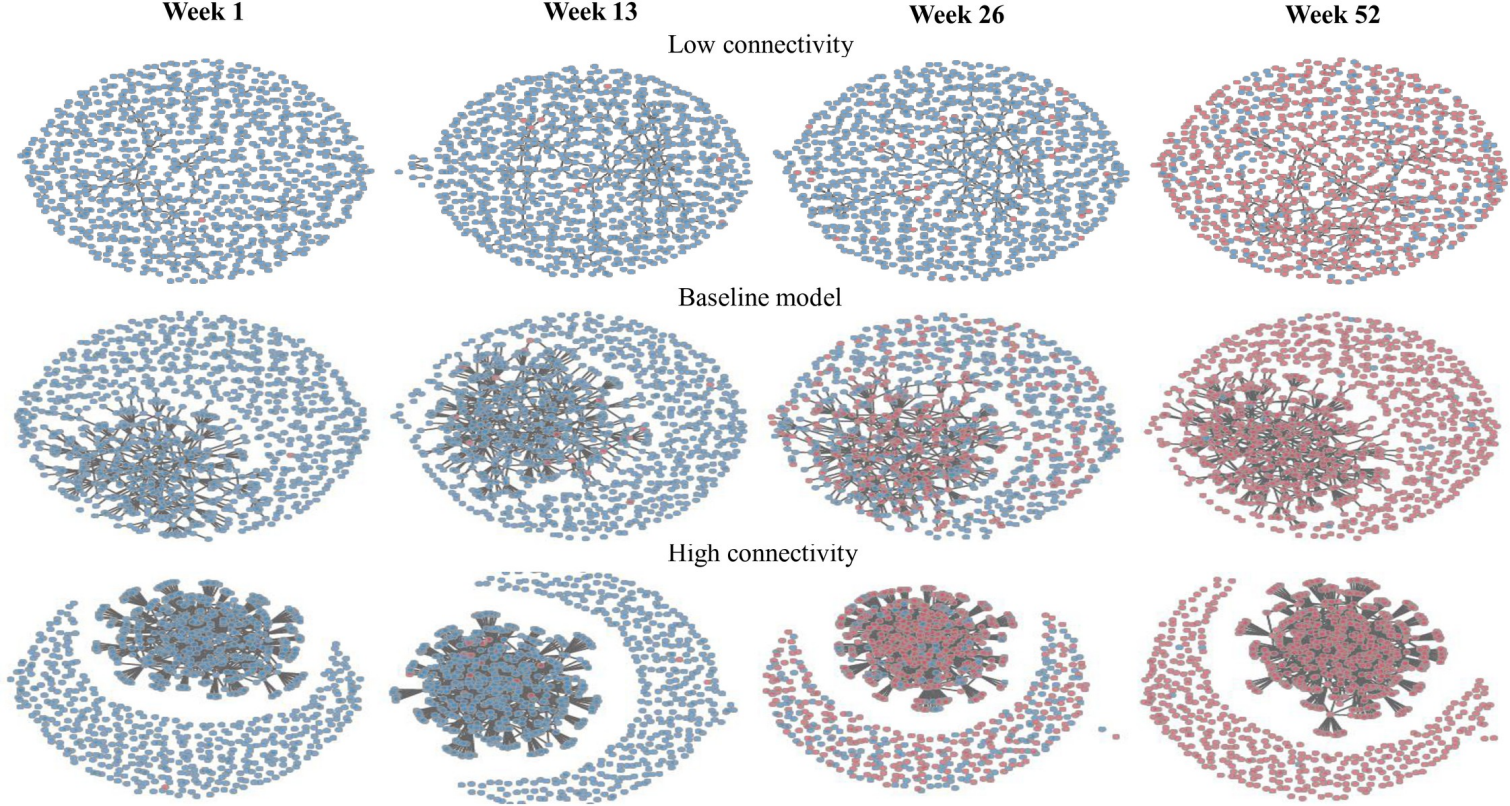
A snapshot of farm networks at different time intervals (week 1, 13, 26 and 52) under the different connectivity scenarios (blue: Susceptible farms and red: Infection farms).

The first SI model simulated the number of infected farms (median, 5 and 95 percentiles) by farm type (Table 3). The three scenarios modelled in this way (baseline, low, and high) evaluated the impact of connectivity on disease transmission. Not surprisingly, the median number of the total infected farms was higher as the connectivity increased (low: 659, baseline: 968 and high: 993). All three scenarios showed that without intervention, the epidemic peaked at week 52. Half of the infected farm were observed at week 32 in the baseline scenario, while for the low and high connectivity simulated scenarios, they were at week 50 and 22, respectively. In addition, we plotted the static networks of farms at four different time points (week 1, 13, 26, and 52) (Fig 1) during the simulation. The blue and red nodes indicated susceptible and infected farms, respectively. It showed that the high connectivity scenario had relatively more infected farms compared to the baseline and low connectivity scenarios at the same time point.

**Table 3:** Median number of infected pig farms and time required to reach the peak epidemic under the change of the overall mean degree.

Scenario	Overall mean degree	Median number of infected farms (5 and 95% percentiles)				Week to peak epidemic
		Overall	Small	Medium	Large	
Baseline	0.75 (375 edges)	968 (579–992)	684 (409–697)	235 (120–246)	50 (50–50)	52
Low	0.5 (250 edges)	659 (83–913)	470 (58–651)	133 (13–212)	49 (11–50)	52
High	1 (500 edges)	993 (889–999)	697 (637–700)	246 (201–249)	50 (50–50)	52

To evaluate the impact of the culling strategy on the number of infected farms, a total of four scenarios were simulated depending on the timing of culling after a farm was infected (Table 4). We found that the timing of culling at 16, 12, 8, and 6 weeks had resulted in a reduction of the number of median infected farms by 81.92%, 91.63%, 100%, and 100%, respectively. In particular, R3 and R4 scenarios (culling at 6 or 8 weeks) showed the largest decrease in the number of infected farms compared to other scenarios. The proportion of iterations with no transmission of the virus beyond the index farm for scenarios R1-4 was 43.0%, 47.0%, 71.0% and 82.5%, respectively.

**Table 4:** Number of median infected pig farms at different culling timings–the same TP (0.6) was applied to all scenarios.

Scenario	Timing of culling	Number of median infected farms (5 and 95% percentiles)				% change in median outcome of all farms compared to baseline
		Overall	Small	Medium	Large	
Baseline	Null	968 (579–992)	684 (409–697)	235 (120–246)	50 (50–50)	N/A
R1	16 weeks	175 (0–317)	127 (0–232)	45 (0–77)	5 (0–22)	-81.92%
R2	12 weeks	81 (0–222)	55 (0–163)	22 (0–49)	1 (0–18)	- 91.63%
R3	8 weeks	0 (0–107)	0 (0–77)	0 (0–22)	0 (0–9)	- 100%
R4	6 weeks	0 (0–42)	0 (0–31)	0 (0–12)	0 (0–4)	- 100%

The sensitivity analysis was conducted for contact rate (Small-medium-large) parameters compared to the baseline scenario (Table 5). A reduction of contact rate by 25%, 50%, and 75% from the baseline scenario showed that the number of infected farms had decreased by 9.29%, 40.81% and 96.28%, respectively. Finally, our evaluation of the implication of stability of ties between farms indicated that if the farms were to have the same trading partners for at least six months could significantly reduce the median number of infected farms to two (95^th^ percentile: 413) than in the basic model in which farms were mostly assumed to trade with new farms.

**Table 5:** Sensitivity analysis of the median epidemic size of simulated ASF outbreaks to changes in contact rates.

Scenarios	Parameters		% change in contact rate	Median epidemic Size (5 and 95 percentiles)	% change in median outcome compared to baseline
	Transmission probability	Contact rate (S, M, L)			
Baseline	0.6	(0.241, 0.236, 0.021)	N/A	968 (579–992)	N/A
CR 1	0.6	(0.181, 0.177, 0.016)	-25%	878 (43–964)	- 9.29%
CR 2	0.6	(0.121, 0.118, 0.011)	-50%	572 (1–859)	- 40.91%
CR 3	0.6	(0.060, 0.059, 0.005)	-75%	36 (1–394)	- 96.28%

## Discussion

To our knowledge, this was the first network simulation model to evaluate the transmission of ASF virus among swine farms in Vietnam. Our modelling exercise provided valuable insight into the importance of incorporating various network structures in the model. The credible parameters for direct and indirect contact rates were utilized from previous data based on the farm survey, which made our network model more realistic. Our results showed that the level of connectivity amongst farms plays a vital role in the spread of disease, which is consistent with previous studies that used network-based models for disease spread [27, 28]. Importantly, Lebl et al. (2016) suggested that trade activities were the most important risk factor for disease transmission. In addition, one study was conducted using the EpiModel package used in the current study to evaluate the consequence of connectivity in the spread of influenza in pigs [27]. Findings showed that high animal movement significantly raised the risk of disease transmission. In Vietnam, each province has some data on livestock movement, but the accuracy and quality of data are questionable, which makes it difficult to track the likely spread of disease. Animal movement patterns across the country must, therefore, be systematically monitored and recorded for disease control and prevention.

Since the first occurrence of ASF was reported in northern Vietnam (called the “Red River Delta” region) in February 2019, the number of reported outbreaks and affected provinces rapidly increased within 4–5 months. A number of potential risk factors have been suggested, such as a long porous border with China and unregulated trading activities; internal travel with ASF infected food; low biosecurity; use of swill feeding; not fully culling all pigs in infected premises, throwing carcasses to public areas, illegal pig slaughtering and the proximity of pig slaughterhouses to main roads. Furthermore, increased human and animal movements in the run-up to the Tet holiday (Vietnamese New Year in February) may have played a critical role in the spread of the disease.

It was assumed that the ASF virus in Vietnam was transmitted from China through pig movement and pork products, or infected fomite [29]. One study found that the ASF virus strain was 100% identical to the circulating strain in China [10]. It was likely that the ASF virus had spread from north to central and southern provinces, following a very similar pattern of outbreaks of the highly pathogenic porcine reproductive and respiratory syndrome (HP-PRRS) in 2007 [30]. At that time, the HP-PRRS virus was first reported in China, then rapidly spread into Vietnam and other Southeast Asian countries [31–33]. One network analysis study also found that pig movements were highly connected, moving across northern, central, and southern provinces in Vietnam [22].

In Vietnam, ASF virus transmission within and between farms has shown both slow and fast transmission patterns. More than 90% of outbreaks have been reported at small- and medium-sized farms, while large commercial farms with high biosecurity have not been affected. The most impacted groups in terms of disease burden have tended to be smallholder farmers, who are unable to maintain the required levels of biosecurity to prevent and mitigate the spread of ASF. Because smallholders are responsible for more than 70% of Vietnam’s pig production, it is a huge challenge to control and prevent the spread of ASF.

We also found that the timing of culling was key to minimizing disease transmission. The proportion of iterations with no transmission increased the earlier the culling strategy was conducted in the models. The rationale for choosing a wider window to implement the culling strategy (starting from six weeks to 16 weeks from occurrence of the first outbreak) was based on expected delays and variability in the time of detection of an outbreak, its notification to the authorities, and preparedness required to start culling. Irrespective of the timing of culling, our results, however, unequivocally demonstrate significant to a complete halt in the spread of the virus. Some other studies have also suggested that the culling of infected herds and movement control are the most effective control strategies [11, 34–36]. Our study also provided evidence that reduced contact rates had an impact on the number of median infected farms. While we did not consider the scenarios for hypothetical vaccination, one study suggests that early combined interventions (vaccines and strengthened biosecurity) would result in a dramatic reduction of pig deaths [37]. The same study emphasizes the importance of early detection in managing ASF outbreaks.

Early detection and risk-based surveillance of diseases are critical for disease control and prevention. However, given the current practices of farmers, early detection and reporting of transboundary emerging diseases (including ASF) are unlikely to happen in Vietnam. In addition, farmers have a tendency to manage issues by themselves, either as a result of a lack of knowledge or trust in outbreak prevention and control policies. This is further exacerbated by low compensation rates, complicated administrative procedures, uncertain timing of distribution of compensation, and improper culling practices by authorities, which can result in the quick spread of the disease across the country.

One key factor that contributed to the uncontrollable spread of the ASF virus in Vietnam was the low biosecurity levels of small and small-to-medium commercial farms (accounting for 80–90% of farms in Vietnam). Another was the limited ability of veterinary services to address outbreaks at commune and village levels. Competencies amongst local animal health professionals can vary vastly across the country and suffer as a result of poor training and low income that is often dependent on the ability of farmers to pay for their services. In addition, the restructuring of district services and the lack of commune animal health workers (CAHW) lead to a total absence of public veterinary services in the field. As a result, animal diseases are rarely diagnosed in laboratories and are either not reported in time, or reported inaccurately, leading to insufficient or inappropriate treatment of animal diseases and contributing to the spread of disease. These challenges would need to be addressed in order to prevent the future spread of infectious diseases in Vietnam.

The main limitation of this study was that we were not able to take into account all possible risk factors and uncertainties associated with estimated parameters for disease transmission in the network models. For example, it was assumed that infected farms were infectious during the study period, but could be overestimated as farmers would conduct intervention activities to eliminate the ASF virus. The direct contact for TP was 0.6 in the network models, which might be an over-estimation as some farms may use the AIAO production system or have relatively high biosecurity measures in place. In addition, we used the same TP (0.6) for direct and indirect contacts to create a new aggregate contact rate for small and medium farms, which were likely to be overestimated. Our estimated mean contact rates through farm survey may not be representative for the entire country as farm systems are slightly different depending on the regions. These values need to be customized for Vietnam based on evidence from field data in future studies. Also, in the absence of reliable outbreak data, predictive ability of the models could not be evaluated and therefore, we cannot make robust quantitative inferences from the results based on our network models. However, the developed models still provide valuable insights on the patterns of outbreaks and the effect of different mitigation strategies on halting the outbreaks. This network model gave the same weight to each edge, which may not be realistic. Some studies suggest that the results can be different when connections with variable weighting are considered in the model [38, 39].

## Conclusions

This study has provided valuable insights into how ASF virus can spread between farms via direct and indirect contacts. It has been a challenge to understand the epidemic dynamics of infectious diseases as various/complex factors are associated with each other. Network analysis proved useful in understanding disease transmission among farms by estimating potential pathways [27, 40]. It opened up opportunities to address the importance of animal movement when developing risk-based surveillance and cost-effective control measures, especially given that systemic records are limited in Vietnam. Our study showed that pig movements among farms had a significant influence on the transmission dynamics of ASF virus. In addition, we found that the either timing of culling, reduction in the number of trading partners each farm had, or decreased mean contact rate during the outbreaks were essential to prevent or stop further outbreaks.

## Supporting information

S1 Fig Transmission of ASF virus from susceptible to infectious via direct/indirect contacts (a) and simple diagram of farm movement stricture in Vietnam (b) (dash arrow: rare movement).
(TIF)

## Abbreviations

ASF: African swine fever
SNA: social network analysis
TP: transmission probability
SI: susceptible-infected
CF: continuous flow
AIAO: All-in-all-out system
SIR: susceptible-infected-Removed
HP-PRRS: highly pathogenic porcine reproductive and respiratory syndrome
CAHW: commune animal health workers
