# Supplementary figures and images for: A stochastic network-based model to simulate farm-level transmission of African swine fever virus in Vietnam
Source: PLoS One. 2021 Mar 3;16(3):e0247770. doi: 10.1371/journal.pone.0247770 (PMC7928462; doi:10.1371/journal.pone.0247770)

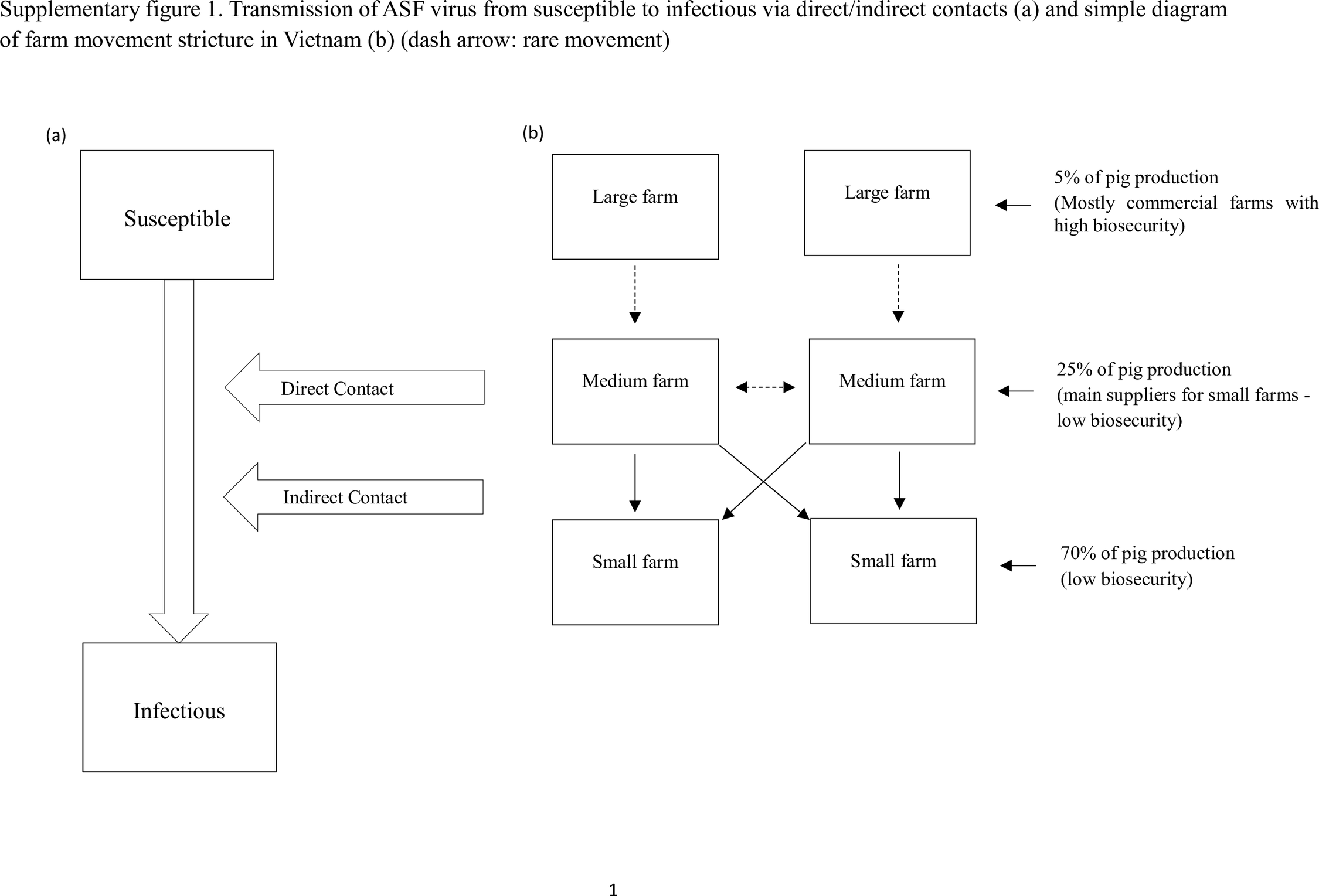

Supplement: S1 Fig — Transmission of ASF virus from susceptible to infectious via direct/indirect contacts (a) and simple diagram of farm movement stricture in Vietnam (b) (dash arrow: rare movement). (TIF) [file pone.0247770.s001.tif]
